# The Effect of Early Adverse Experiences on Coping Strategies, Mental Health, Brain and Cognition

**DOI:** 10.1002/alz70860_097317

**Published:** 2025-12-23

**Authors:** Morgane Kuenzi, Delia A Gheorghe, Sarah Bauermeister

**Affiliations:** ^1^ University of Oxford, Oxford, United Kingdom; ^2^ Transylvanian Institute of Neuroscience, Cluj‐Napoca, Cluj, Romania; ^3^ University of Oxford, Oxford, Oxfordshire, United Kingdom

## Abstract

**Background:**

Literature has shown the detrimental effects of the experience of early adversity on later‐life mental health, brain, and cognition. However, these associations are complex, and the underlying mechanisms are not yet understood. Consequently, identifying how early adverse experiences impact mental health, brain, and cognition tackles the difficult question of mechanisms. Shedding light on such explanatory pathways may help promote healthy brain and cognitive aging by identifying and intervening on the risk and protective factors.

**Method:**

This study aimed to investigate the effects of early adversities and the mediating roles of coping strategies and neuroticism on mental health, brain, and cognition. The UK Biobank dataset (*N* = 502,363, *M_age_* = 58, *SD_age_* = 8.09) including behavioral and imaging data was used and combined in a structural equation model (SEM).

**Result:**

Results highlighted different effects according to the type of early adversity experienced. Only physical neglect was associated with cognition and none of the early adversities had a direct effect on brain volume. However, significant mediations through some of the coping strategies were found between early adversity and cognition and between early adversity and brain volume.

**Conclusion:**

Our results are in line with the literature findings showing associations between early adversity and mental health, brain, and cognition. Importantly, this study highlights the importance of personality traits and coping strategies for these associations, showing that actively working on these coping strategies may buffer the negative impact of early adversity on mental health, brain, and cognition and promote healthy aging

